# Supplementary material for: Impact of the early COVID-19 pandemic on outcomes in a rural Ugandan neonatal unit: A retrospective cohort study
Source: PLoS One. 2021 Dec 16;16(12):e0260006. doi: 10.1371/journal.pone.0260006 (PMC8675646; doi:10.1371/journal.pone.0260006)
Supplement: S1 Table — (DOCX) [file pone.0260006.s001.docx]

**S1 Table. Impacts on Maternal Child and Health Reported Globally during the Early COVID-19 Pandemic and their Experience at Kiwoko Hospital**

| **Global Trends Reported** | **Kiwoko Hospital Experience** |
| --- | --- |
| **Maternity** | |
| Restricted transport for mothers and babies(1-4) | Early lockdown restrictions in April involved an inability for pregnant women and new mothers to seek care via hiring a ride on a motorcycle, or via minibus- the most common and accessible forms of transportation. There were multiple police checkpoints and people feared punishment if found on the road. In addition, early lockdown guidelines required that people could only travel with approval from the resident district commissioners (RDCs), even in a medical emergency. Unfortunately, each district has only one RDC, making it difficult for pregnant women to get travel clearance. This resulted in many delays, and this measure was soon removed. However, confusion persisted about who was allowed to travel for medical care.  Lockdown also restricted travel between districts, this also affected ability to discharge antenatal patients if they lived out-of-district.  Once restrictions eased there was more availability of ambulances free-of-charge to take mother or baby to next level of care, due to recent funding to support referral system. Once transportation resumed, it was at half capacity, which increased prices and made travel still prohibitive for many. |
| Decreased antenatal care(1, 4-7) | Antenatal care clinic attendance was **decreased** at Kiwoko Hospital 8% during the early COVID-19 period compared to this period one year previous. This was especially true during the lockdown of April/May when appointments of the low-risk mothers were de-prioritized.  Some pregnant women moved near hospitals as they knew it was going to be difficult to travel when needed. |
| Increased adolescent pregnancy(4, 8) | The number of women under the age of 19 who gave birth at Kiwoko Hospital was **unchanged** during COVID-19 [13% down from 14% pre-COVID].  Among neonatal unit admissions, mothers were one year younger on average, but no increase in proportion of teenage mothers. |
| Decreased facility birth(1, 3, 6, 7, 9-12) | The Kiwoko Hospital maternity unit had a 14% **decrease** in admissions and 13% decrease in births during the early COVID, compared to the same period one year prior. |
| Decreased cesarean sections(8) | Kiwoko Hospital performed a **higher rate of cesareans** during the COVID-19 period than previously (39% vs. 31%).  The maternity unit reports subjectively marked increase in women who arrived in obstructed labor. |
| Increased births at home or with traditional birth attendant(13) | Kiwoko Hospital’s Maternity unit reports subjectively **increased** admissions following home births of mothers who did not feel they could safely pass all the security checkpoints in the roads during lockdown.  NICU data in this report shows among admitted newborns they were less likely to have been born at home (9.5% vs. 11.5%) but more likely to have been born via traditional birth attendance (4.2% vs. 3.4%). |
| Decreased availability of labor medications (5, 14) | The maternity unit experienced **decreased availability** of oxytocin and methyldopa. |
| Increased stillbirth(5, 8, 15) | Kiwoko Hospital **stillbirth rate decreased** during the early COVID-19 period to 33.1 per 1,000 births from 39.3 previously.  Unit leadership, however, report 8 stillbirths in one particularly bad month of the pandemic, up from a typical of 2-3 per month. |
| **Neonatal Unit** | |
| Decreased neonatal intensive care admissions(5, 16, 17) | Admissions decreased in the early COVID-19 pandemic by 9%. |
| Decreased outborn admissions(10, 18) | Proportion of admissions that were outborn decreased to 46% during COVID-19 from 52% in previous period. |
| Increased preterm birth(5, 16) | Kiwoko had increased rates of low birthweight admissions (58% up from 54%) among inborn patients, but decreased among outborns (45% down from 50%).  Staff perceive that mothers at high-risk of preterm delivery come to stay near Kiwoko for “state of the art” care after delivery. |
| Increased birth asphyxia(16) | Kiwoko Hospital experienced increased both rates and absolute numbers of admissions for asphyxia during COVID-19 period compared with previous [22% (127/567) up from 15% (93/619)] and this trend held among both inborn and outborn patients. |
| Decreased kangaroo mother care (KMC)(10, 19) | The unit was able to continue KMC and primarily uses electric methods for thermoregulation. |
| Decreased blood supply(1) | Decreased supply of blood occurred as main donors (nursing students) were home because schools had been closed due to the pandemic. |
| Earlier discharge requested(10) | The neonatal unit had no change to discharge practices. Staff report some families were more anxious for discharge, however. |
| Increased facility neonatal mortality(11, 17, 20) | Increase in neonatal unit mortality from 11% pre-COVID reference period to 16% during April to October 2020 reported in this study. |
| Decreased facility-based infant follow-up(10, 21) | Follow-up clinic was closed temporarily early in the COVID-19 period. However, at home follow-up of at-risk infants continued through a “hospital to home” program, with 738 infants (94% of all babies who were eligible for the program) receiving at least one home-follow up visit from a trained community health worker during 2020.(22) |
| **Parents** | |
| Decreased maternal support by family(10) | Mothers may have been less supported (socially, financially and with food) as less family were allowed in the hospital due to changes to visitor policies during the pandemic. |
| Difficulty with medical expenses/ food security(1, 4, 17, 23) | In the maternity unit, mothers had more difficulty paying their bills resulting in delayed discharge and congestion in the unit.  In the neonatal unit, mothers are given accommodation and meals during their stay. The neonatal unit/hospital fee is heavily subsidized and waivable if someone is unable to pay. |
| **Staff and Community** | |
| Restricted personal protective equipment (PPE)(1, 3, 10) | Maternity and neonatal units report they had the PPE they needed. |
| Decreased staffing levels(1, 10, 24, 25) | Kiwoko nurses and doctors generally live on-campus and were able to get to work during lockdown. At one point, staffing in the maternity unit decreased from 30 midwives down to 23 due to requirements for quarantine after a COVID-19 exposure. |
| Staff experience/ COVID fear(10, 24) | Nurses report no perceived COVID risk in taking care of neonatal patients.  Supervisors had concern for neonatal and maternity unit staff depression due to increased workload, inability to visit their families due to travel restrictions, and the challenge of dealing with excess mortality in the newborn unit.  There was a general fear of testing positive for COVID-19 at the hospital, because if anyone tested positive they would be taken away by a team in hazardous material suits to a government facility for quarantine. |
| Community fear of COVID at facilities and/or belief the facility was closed(6, 7, 10) | There was a period when staff tested positive for COVID-19 and people heard “Kiwoko had COVID”. There was significant stigma and women drew back from coming to the facility. Anyone who was tested for COVID-19 (whether positive or negative) was stigmatized. |
