# Supplementary material for: Impact of the early COVID-19 pandemic on outcomes in a rural Ugandan neonatal unit: A retrospective cohort study
Source: PLoS One. 2021 Dec 16;16(12):e0260006. doi: 10.1371/journal.pone.0260006 (PMC8675646; doi:10.1371/journal.pone.0260006)
Supplement: Attachment — Submitted filename: 21.docx [file pone.0260006.s004.docx]

PONE-D-21-20950

Impact of the early COVID-19 Pandemic on Outcomes in a Rural Ugandan Neonatal Unit: a Retrospective Study

PLOS ONE

**Response to Reviewers**

**Reviewer #1**: by Ermias Sisay Chanie /MSc in pediatrics and Child health Nursing)

Debre Tabor University, Debre Tabor, Ethiopia

Thanks for give an opportunity to plos one chief editors to reviewing this article. This research is important on reducing neonatal mortality and morbidity in worldwide in general and in study ara in particular, in the meantime it is one of the international concerns. African countries including Ugandan works to reduce the mortality of neonate through monitoring and evaluation the ‘’outcomes neonate in Neonatal Unit”.

Minor

1. General English: The review recommends the article be copyedited to improve language and grammar used

We appreciate this recommendation. Our goal is to be grammatically accurate. The English language/grammar use has been edited where appropriate. If the editors have further suggestions for optimizing grammar we are open to them.

2. Check the table number frequency, percentage and p- value for each column

These have been checked, please see discussion of p values in response to reviewer 2 below.

Major concern

Title

1. In my point of view the title better to modified based on your conclusion, since it focuses on the impact of COVID-19 in “maternal and newborn demand for, access to and quality of peripartum healthcare’’ etc.

The title has been modified to “Impact of the early COVID-19 Pandemic on Outcomes in a Rural Ugandan Neonatal Unit: a Retrospective Cohort Study”. Although we agree with the reviewer that the impact on “maternal and newborn demand for, access to and quality of peripartum healthcare” is important we did not have any direct measures of this so do not feel comfortable including it in the title.

2. Rural vs Neonatal Unit? I am not clear with it. I am not expected neonatal unit in rural area.

As the reviewer notes, it is unusual, however Kiwoko hospital is a secondary neonatal unit in a rural area. Due to its clinical reputation, it has become a referral unit for three districts in central Uganda.

Introduction

1. In my view, better to focus on your objective, which mean the effect of COVID-19 on maternal health, or neonatal health or healthcare system in general

The introduction text has been updated to focus on the effect on maternal/neonatal health and the healthcare system in general:

*“An early survey by the WHO reported 80% of countries had disruptions to essential health services and these were exacerbated in lower income countries. Little has been published, however, on low resource facility based neonatal outcomes from low resource facilities during the COVID-19 pandemic, nor describing experiences of peripartum care availability.^11-14^ “*

2. Try to show any attempting solution to reduce the burden of COVID-19 related to your objective in the study rea

We added to the introduction: “*During the early COVID-19 pandemic, this facility was able to obtain personal protective equipment for staff and maintain neonatal care but peripartum mothers had difficulty traveling to the facility due to lockdown.*”

3. The identified gaps before conducting the study? And the implication of the study to fill the appreciated gaps

We added *“Pandemic control measures affecting access to newborn care remain highly dynamic and no country-level data of neonatal survival during this period are available.”*

Methods

1. Discuss about your study populations vs source population of the study participant clearly

We added *“****Study Population-*** *Included in this study are* *patients admitted to the Kiwoko Hospital (KH) neonatal unit during the period of study. There were no exclusion criteria.” The “setting” section goes on to describe the type patients admitted to this unit and the typical provision of care in the unit.*

2. your Inclusion criteria vs Exclusion criteria of the study participant clearly

See added text in response to #1 above re: inclusion/exclusion criteria.

3. Better to included data set in statical analysis and try to explore about descriptive statics that was used etc.…?

The dataset is now uploaded in accordance with journal requirements. We agree with the reviewer and focused on descriptive statistics in this report. If further suggestions can be considered, please clarify these.

4. Adding about goodness of fit and model fitness?

We did not use a model in this analysis.

**Reviewer #2:**

Abstract is too long and should not exceed 300 words for The PLOS ONE. Please describe clearly the main objective of this study and summarize the most important results and their significance.

Thank you for this guidance. The abstract is now < 300 words. It has been revised to better describe the objective and most important results and their significance.

Introduction:

The overall aim of this study and whether that aim was achieved are missing.

The introduction has been revised to better describe the aim:

*“The aim of this study is to describe this facility’s experience with providing peripartum care during the pandemic by utilizing this longitudinal dataset to explore changes to facility-based neonatal outcomes before and during the early phase of the COVID-19 pandemic.”*

Methods:

The role of the funding source should not be in the method section

It has been removed.

The statistical analysis to compare the groups are missing or incorrect (proportion tests?)

As suggested by the reviewer, we used a proportion test to compare the groups. This is described in the methods:

*“****Statistical analysis***

*The mortality rate pre and during the early COVID-19 period was computed as a proportion of infants who died to the total number of infants admitted to the unit in the same period. To determine the potential impact of the COVID-19 pandemic on neonatal mortality in the unit, we computed the relative change in mortality rate between two time periods of the same months for the 2 sequential years. Proportion tests were used to compare the rates between two time periods of the same months. “*

Results:

Fig 1. It should clarify the term “outborn” and “inborn” in the legend

The figure legend has been updated with:

“Outborn” refers to patients born outside KH. “Inborn” includes those born at KH.”

Table 2. the “+-“ should replace the points in all numbers. Same for the text across the manuscript

p-values should be added to the table. The statistical analysis should be described in the table.

- The decimal points have been revised throughout the tables and manuscript
- We appreciate the comment and have discussed among our authors but respectfully disagree with the style of including p values in table 2 which describes demographic characteristics in the two time periods. P values may be misleading in comparing groups from two different periods given the seasonal nature of neonatal mortality and would be at risk of type 2 errors.

If the editorial team feels a formal comparison of the demographics is important to our manuscript, we would ask to change this table to a comparison of seasonally matched periods (ie comparing April to September 2020 with April to September 2019). However, we feel the most important data to show is immediately prior to the COVID-19 pandemic. Formal appropriate comparisons of mortality adjusting for seasonal trends are later in the manuscript.

Figure 2 legend should describe the sample size and the statistical analysis used. It should not repeat the results of the figure. It also should specify the term inborn and outborn.

- FIgure 2 legend now:
  - no longer repeats the results of the figure
  - includes *“1,234 patients were outborn (born outside Kiwoko hospital) and 1,260 born at Kiwoko (inborn) during the periods of study.“*
  - includes *“Excess mortality is the amount of mortality seen above what would be expected for trend, calculated as the relative difference between periods divided by mortality in the reference period.”*

While revising your submission, please upload your figure files to the Preflight Analysis and Conversion Engine (PACE) digital diagnostic tool, <https://pacev2.apexcovantage.com/>. PACE helps ensure that figures meet PLOS requirements. To use PACE, you must first register as a user. Registration is free. Then, login and navigate to the UPLOAD tab, where you will find detailed instructions on how to use the tool. If you encounter any issues or have any questions when using PACE, please email PLOS at figures@plos.org. Please note that Supporting Information files do not need this step.

This has been done.

**Additional Journal Requirements:**

1. Please ensure that your manuscript meets [PLOS ONE's style requirements](https://journals.plos.org/plosone/s/file?id=wjVg/PLOSOne_formatting_sample_main_body.pdf), including those for file naming.

The manuscript has been updated to comply with the PLOS ONE style requirements.

2. In ethics statement in the manuscript and in the online submission form, please **provide additional information about the database used in your retrospective study**. Specifically, please ensure that you have discussed whether all data were fully anonymized before you accessed them and/or whether the IRB or ethics committee waived the requirement for informed consent. If patients provided informed written consent to have their data used in research, please include this information.

The ethics statement was revised to “*The data were fully anonymized before accessed by the research team and the ethics committee waived the requirement for informed consent*.”

3. Thank you for stating the following in the Financial Disclosure section: "Data collection and data analysis (PM) was supported by **Adara Development** (<https://www.adaragroup.org/>). Adara development did not influence the study design, data analysis/interpretation, information reported nor decision to publish. "

We note that one or more of the authors have an affiliation to the commercial funders of this research study: Adara Development.

Thank you for the opportunity to clarify this. See responses to 3.1 and 3.2 below.

3.1. Please provide an **amended Funding Statement** declaring this commercial affiliation, as well as a statement regarding the Role of Funders in your study. If the funding organization did not play a role in the study design, data collection and analysis, decision to publish, or preparation of the manuscript and only provided financial support in the form of authors' salaries and/or research materials, please review your statements relating to the author contributions, and ensure you have specifically and accurately indicated the role(s) that these authors had in your study. You can update author roles in the Author Contributions section of the online submission form.

Please also include the following statement within your amended Funding Statement.

“The funder provided support in the form of salaries for authors [insert relevant initials], but did not have any additional role in the study design, data collection and analysis, decision to publish, or preparation of the manuscript. The specific roles of these authors are articulated in the ‘author contributions’ section.”

If your commercial affiliation did play a role in your study, please state and explain this role within your updated Funding Statement.

On further review, Adara development does not fit criteria to be a funder in the study. We have reviewed and updated the statements relating to the author contributions in the manuscript.

The funding statement (emailed to plosone@plos.org) now includes “No outside funding was provided for this study.”

3.2. Please also provide an **updated Competing Interests Statement** declaring this commercial affiliation along with any other relevant declarations relating to employment, consultancy, patents, products in development, or marketed products, etc.

Within your Competing Interests Statement, please confirm that this commercial affiliation does not alter your adherence to all PLOS ONE policies on sharing data and materials by including the following statement: "This does not alter our adherence to PLOS ONE policies on sharing data and materials.” (as detailed online in our guide for authors http://journals.plos.org/plosone/s/competing-interests). If this adherence statement is not accurate and there are restrictions on sharing of data and/or materials, please state these. Please note that we cannot proceed with consideration of your article until this information has been declared.

Please respond by return email with an updated Funding Statement and Competing Interests Statement and we will change the online submission form on your behalf.

Please know it is PLOS ONE policy for corresponding authors to declare, on behalf of all authors, all potential competing interests for the purposes of transparency. PLOS defines a competing interest as anything that interferes with, or could reasonably be perceived as interfering with, the full and objective presentation, peer review, editorial decision-making, or publication of research or non-research articles submitted to one of the journals. Competing interests can be financial or non-financial, professional, or personal. Competing interests can arise in relationship to an organization or another person. Please follow this link to our website for more details on competing interests: http://journals.plos.org/plosone/s/competing-interests

The updated funding and competing interests statement has been supplied by return email as requested.

4. We note that you have indicated that data from this study are available upon request. PLOS only allows data to be available upon request if there are legal or ethical restrictions on **sharing data publicly**. For information on unacceptable data access restrictions, please see <http://journals.plos.org/plosone/s/data-availability#loc-unacceptable-data-access-restrictions>.

In your revised cover letter, please address the following prompts:

a) If there are ethical or legal restrictions on sharing a de-identified data set, please explain them in detail (e.g., data contain potentially identifying or sensitive patient information) and who has imposed them (e.g., an ethics committee). Please also provide contact information for a data access committee, ethics committee, or other institutional body to which data requests may be sent.

b) If there are no restrictions, please upload the minimal anonymized data set necessary to replicate your study findings as either Supporting Information files or to a stable, public repository and provide us with the relevant URLs, DOIs, or accession numbers. Please see <http://www.bmj.com/content/340/bmj.c181.long> for guidelines on how to de-identify and prepare clinical data for publication. For a list of acceptable repositories, please see <http://journals.plos.org/plosone/s/data-availability#loc-recommended-repositories>.

We will update your Data Availability statement on your behalf to reflect the information you provide.

The dataset has been uploaded as a supplementary table.
